# Plant immune receptor decoy: Pathogens in their own trap

**DOI:** 10.18632/oncotarget.4717

**Published:** 2015-07-01

**Authors:** Alice Delga, Clémentine Le Roux, Laurent Deslandes

**Affiliations:** INRA, Laboratoire des Interactions Plantes-Microorganismes (LIPM), Castanet-Tolosan, France

**Keywords:** Chromosome Section, RPS4, RRS1-R, PopP2, Avr-Rps4

Microbial pathogens have evolved sophisticated strategies to infect their hosts, often resulting in disease. The host, in turn, can produce novel proteins (receptors or antibodies) that recognize pathogen molecules to trigger defense. Unlike animals, plants do not possess any adaptive immunity to defend themselves against pathogens. Therefore, they rely entirely on their genetic resistance capability (innate immunity) conferred by a family of receptors expressed in individual cells. The plant innate immune system can be divided into two layers of defense. The first, known as pattern-triggered immunity (PTI) leading to basal defense, involves the recognition of microbe-associated molecular patterns (MAMPs) by corresponding plasma membrane pattern-recognition receptors (PRRs). PTI can be suppressed by specific pathogen virulence factors (known as effectors). To detect such pathogen molecules or their interference with host proteins, plants have evolved a second layer of defense, known as effector-triggered immunity (ETI) [1]. ETI is mediated by intracellular nucleotide-binding–leucine-rich repeat receptors (NLRs) that resemble mammalian NLRs [2]. The speed with which microbial populations can produce new effectors places enormous pressure on plant hosts to fight back with genetically new or altered receptor recognition modes.

Le Roux *et al*. and Sarris *et al*. (2015) recently described in *Cell* an exquisite immune receptor decoy mechanism in the model plant species *Arabidopsis* in which a potent bacterial virulence activity is turned into defense gene activation [3, 4]. Plant NLRs often function in pairs in which one or both members contain different protein domains of unknown relevance. This is the case for a pair of *Arabidopsis* NLRs, Resistance to *Pseudomonas syringae* 4 (RPS4) with Resistance to *Ralstonia solanacearum* 1 (RRS1-R), that cooperate genetically and molecularly to detect PopP2 and AvrRps4 effectors from root-infecting *Ralstonia solanacearum* and leaf-infecting *Pseudomonas syringae* bacteria, respectively. Unlike RPS4, RRS1-R contains at its carboxyl terminus a conserved ‘WRKY’ DNA-binding domain of plant WRKY transcription factors that orchestrate biotic stress responses by recognizing W-box motifs in gene promoters. Molecular and structural analyses of RRS1-R/RPS4 interactions suggest that both receptors associate to form an inhibited, pre-activation receptor complex that is activated upon direct binding of effectors [5]. Because PopP2 and AvrRps4 interact with RRS1-R and PopP2 acetyltransferase activity is necessary to trigger RRS1-R/RPS4 immunity [6], RRS1-R was hypothesized to serve as both an inhibiting molecule of the NLR pair at the DNA in uninfected plants and a direct sensor of PopP2 and AvrRps4 upon infection. How this NLR complex perceives these two unrelated effectors remained enigmatic.

Le Roux *et al*. and Sarris *et al*. (2015) identified the WRKY domain of RRS1-R as a target of PopP2 and AvrRps4. A catalytically active form of PopP2 was found to directly acetylate a key lysine residue (K1221) in the invariant WRKY DNA-binding domain of RRS1-R. Homology modelling predicts that K1221 acetylation disrupts WRKY domain electrostatic potential at the interface with DNA. Consistent with this, by using a FRET-FLIM (Fluorescence Resonance Energy Transfer– Fluorescence Lifetime Imaging Microscopy) approach dedicated to the detection of protein-DNA associations *in situ*, Le Roux *et al.* (2015) found that PopP2 acetylation disables RRS1-R DNA-binding in plant nuclei. Mimicry of RRS1-R acetylation by an acetyl-mimic variant of RRS1-R (RRS1-R^K1221Q^) transgenically expressed in *Arabidopsis* activates RPS4-dependent immunity, indicating that PopP2 acetylation of RRS1-R is a trigger for activation of the NLR pair.

**Figure 1:**
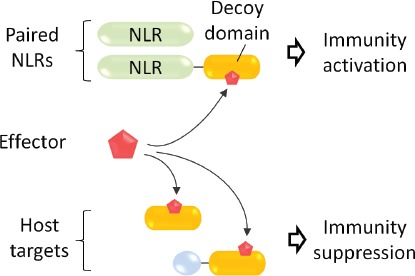
NLR receptors (in green) fused with decoy domains (in orange) that mimic virulence targets of effectors (in red) enable the host to efficiently detect potent virulence activities

From the pathogen angle, PopP2 and AvrRps4 immune-eliciting function are costly since they trigger activation of the receptor complex, which results in ETI. Both groups showed that PopP2 employs the same lysine acetylation strategy to target multiple defense-promoting WRKY transcription factors. In the absence of RRS1-R/RPS4 recognition, PopP2 acetylation dislodges WRKY proteins from their DNA-binding sites and disables their trans-activating functions needed for defense gene expression. This essentially dampens host basal resistance, favouring pathogen invasion. AvrRps4 also interact with other WKRY proteins, suggesting an interference with their defense-related functions [4].

Both studies propose that the WRKY domain in RRS1-R represents an effector target ‘decoy’ which betrays the defense-suppressing abilities of PopP2 and AvrRps4 on their operational virulence targets, the defensive WRKY transcription factors (Figure 1). The direct integration of a WRKY decoy domain within the RRS1-R/RPS4 receptor complex creates an effective ‘radar’ for potent bacterial virulence activities which cannot be easily dispensed with by the pathogens.

The observed fusion of further potential effector target decoy domains with NLR receptors in different plant species suggests a fundamental mechanism in plants for increasing receptor recognition ‘space’ [7]. It is possible that animal immune receptors also integrate decoy domains as molecular mimics of virulence targets of animal pathogens.
